# Poricoic acid A induces apoptosis and autophagy in ovarian cancer via modulating the mTOR/p70s6k signaling axis

**DOI:** 10.1590/1414-431X2021e11183

**Published:** 2021-10-18

**Authors:** Rui Ma, Zhenhua Zhang, Jin Xu, Xueqi Liang, Qiang Zhao

**Affiliations:** 1 Department of Pharmacy, The 305 Hospital of PLA, Beijing, China; 2 Medical School of Chinese PLA, Beijing, China; 3 Department of Gastroenterology, The 305 Hospital of PLA, Beijing, China; 4 Department of Neurology, Chang Zheng Hospital, Navy Medical University, Shangai, China; 5 College of Life Science, Zhejiang Chinese Medical University, Hangzhou, China

**Keywords:** PAA, Poria cocos, Ovarian cancer, mTOR signaling pathway, MHY1485

## Abstract

Due to the high mortality and rapid disease progression, ovarian cancer remains one of the most common malignancies threatening the health of women. The present study was conducted to explore the anticancer effects and the underlying mechanisms of poricoic acid A (PAA), the main components of *Poria cocos*, on ovarian cancer. We investigated the anticancer effects of different concentrations of PAA in the SKOV3 cell line. Cell viability and proliferation were examined by CCK-8 assay. Cellular migration and invasion were assessed by the scratch and Transwell migration assays, respectively. The effect of PPA on cell apoptosis was measured by flow cytometry and caspase-3/8/9 colorimetric assay. Western blot was performed to detect protein level changes related to apoptosis and mTOR signaling pathways. The *in vivo* anticancer effect of PAA was evaluated using xenograft tumorigenesis model in nude mice. Our results showed that PAA suppressed SKOV3 cellular viability, migration, and invasion in a dosage-dependent manner. Flow cytometry results demonstrated PAA treatment could induce SKOV3 cell apoptosis. In addition, increased ratio of LC3-II/LC3-I (a marker for autophagosome formation) was observed after PAA treatment, as well as inhibition of m-TOR and p70s6k phosphorylation. In nude mice, PAA treatment reduced the xenograft tumor weight by 70% (P<0.05). In conclusion, our data suggested that PAA induced apoptosis and autophagy in ovarian cancer via modulating the mTOR/p70s6k signaling axis.

## Introduction

Ovarian cancer is one of the most common life-threating cancers in women. Globally, almost 300,000 women are diagnosed with ovarian cancer every year, which leads to roughly 180,000 deaths (1). The prevalence of ovarian cancer increases with aging. The estimated incidence is 6.6% (per 100,000 women) below the age of 40 and the incidence rises to 48.6% (per 100,000 women) above the age of 65 (2,3). Other risk factors include hereditary mutations, pregnancy history, fertility or infertility drug usage, tubal ligation history, diabetes mellitus, and body mass index (4). The pathology of ovarian carcinogenesis remains unclear. Current theory postulates that recurrent ovulation would cause cyclical injuries and the insufficient repair of the epithelium cells may contribute to ovarian tumorigenesis.

*Poria cocos* is a traditional edible fungus that is also named “Fuling”. The medicinal history of *Poria cocos* can be traced back to thousands of years ago in China (5). In traditional Chinese medicine, *Poria cocos* is considered to be beneficial to metabolic functions of kidney and spleen, and is also used to treat epilepsy. Recent studies have revealed a wide spectrum of biological effects of *Poria cocos*, such as anticancer and ant-inflammatory effects (6,7). Mass spectrometry and pharmacological analyses suggest that the major bioactive component of *Poria cocos* is polysaccharide, which shows multiple bioactivities including anti-tumor, anti-inflammation, anti-aging, anti-oxidation, immunomodulation, and anti-hepatitis effects (6,7). In addition, the “*Poria cocos* polysaccharide oral solution” was developed as a formulated drug and was approved by the Chinese Food and Drug Administration for treating different kinds of cancers, hepatitis, and other diseases (8). There are also other small molecule ingredients of *Poria cocos* that are reported to show bioactive effects, such as chitin, β-pachyman, pachymic acid, tumulosic acid, eburioic acid, pinicolic acid, and triterpene carboxylic acid (8). Despite the widely reported beneficial effects, the major bioactive components in *Poria cocos* contributing to its anticancer effect remain to be elucidated.

Poricoic acid A (PAA) is one of main triterpenoid compounds found in *Poria cocos* extract. Previous studies have demonstrated an antifibrotic effect of PAA *in vivo* and *in vitro* (9,10). PAA treatment seems to regulate multiple important signaling pathways including NF-κB, PI3K/Akt, and TGF-β (11-13). For example, *Poria cocos* polysaccharide (PCP) intake significantly elevates the cytokine levels of IL-2, IL-6, IL-17A, and TNF-α, which inhibit Lewis lung carcinoma tumor growth via the TLR4/TRAF6/ NF-κB signaling axis in mice (11). Although the anti-tumor effects of PAA are reported in certain type of cancers, its activity on ovarian cancer remains to be studied. In this study, we explored the anticancer effects and the potential mechanisms of PAA on ovarian cancer.

## Material and Methods

### Reagents

PAA (10 mM in dimethyl sulfoxide (DMSO)) was purchased from ChemFaces (CFB92838, China) and it was diluted to working concentration in phosphate buffer saline (PBS) before using. MHY1485 was purchased from Sigma (SML0810, USA) and mixed with PAA to a final concentration of 2 μM in PBS before using.

### Cell culture

SKOV3 cells (National Infrastructure of Cell Line Resource of China) were cultured in cell culture medium at 37°C with constant humidity and 5% CO_2_ in a cell culture incubator. Cells were sub-cultured every 2-3 days. The complete cell culture medium contained RPMI-1640 medium (Sigma, USA) supplemented with 10% fetal bovine serum (FBS) (Gibco BRL, USA) and 1% penicillin-streptomycin (100 U/mL) (Caisson, USA).

### Cell counting kit-8 (CCK-8) assay

Cellular viability of SKOV3 cells was determined using a CCK-8 assay kit following manufacturer protocol (Dojindo Molecular Technologies, Japan). In brief, approximately 1×10^4^ cells were seeded onto 96-well plates and incubated overnight at 4°C. Different concentrations of PAA diluted in DMSO (0/30/50/80 μg/mL) were added to the cell culture medium. After 24-h treatment, cells were incubated with 0.5 mg/mL CCK-8 reagent (Solarbio, China) for 3 h at 37°C. The culture media were removed and 100 μL of DMSO were added to each well for 10 min incubation. The absorbance was measured at 570 nm using BioTek ELx800 microplate reader (BioTek, USA).

### Wound healing assay

Approximately 1×10^6^ cells were seeded per well in a 24-well plate. After 24 h, a scratch wound was created using a sterile 200-μL pipette tip in the central region of each well. The cell culture media was then replaced with media containing different concentrations of PAA, and the cells were incubated at 37°C for 24 h. Cell images before and after treatment were captured using an inverted light microscope (Leica, Germany). The migration distance was analyzed using ImageJ software (NIH, USA). The migration rate was calculated as ratio of wound width at 24 h/wound width at 0 h.

### Transwell migration and invasion assay

For the cell migration assay, cells were trypsinized and centrifuged at 800 *g* for 5 min at 4°C. Cell pellets were resuspended in serum-free RPMI-1640 medium and cell number was counted using a hemocytometer (Sigma, Germany) with trypan blue staining. Next, 1×10^5^ cells were resuspended in 200 μL of serum-free medium and added to the upper Transwell chamber. RPMI-1640 (500 μL) medium containing 20% FBS was added to the lower chamber as chemokines. The upper chamber was loaded onto the lower chamber and was incubated for 24 h. Afterwards, 4% paraformaldehyde was added to the upper chamber to fix the cells for 15 min. After fixation, cells were stained with 1% crystal violet for 5 min, and cells not passing through the Transwell membrane were wiped off with cotton swabs. Cells migrated through the Transwell membrane were photographed.

For the invasion assay, Matrigel was prepared by mixing with medium at a ratio of 1:4. The diluted Matrigel (50 μL) was placed onto the Transwell chamber, which was followed by 24-h incubation. Next, 200 μL of serum-free medium containing 1×10^5^ cells was added to the upper layer of Matrigel in the chamber. The remaining procedures of cellular fixation, staining, and imaging were the same as for the cell migration assay.

### Apoptosis assay by flow cytometry

Annexin-V-FITC/propidium iodide (PI) assay was employed for examining apoptosis. SKOV3 cells were seeded at a density of 0.3×10^6^ cells per well in a 6-well plate for 24 h. Then, different concentrations of PAA (0/30/50/80 μg/mL) were added to treat cells for another 24 h. Cells were trypsinized and resuspended in 1x annexin-V binding buffer. The resuspended cells were stained with PI (Solarbio) and annexin V-FITC (Solarbio) for 15 min in the dark at room temperature before flow cytometry analysis.

### Caspase activity assay

Caspase activity assays were performed using the following kits from Abcam (UK): Caspase-3 Assay kit (Colorimetric; ab39401) and Caspase-9 Assay kit (Colorimetric; ab65608). Briefly, 0.3×10^6^ cells/well were seeded onto a 6-well plate and treated with different concentrations of PAA for 6 h. Cells were trypsinized and resuspended in 50 μL of chilled cell lysis buffer for 10 min incubation on ice. After 1 min centrifugation at 10,000 *g* and 4°C, the supernatant was collected. The protein concentration in the cell lysate was measured and 50 μg of proteins were mixed with the reaction mix prepared according to the manufacturer's instructions. The mixture was incubated at 37°C for 1 h and the colorimetric product was measured at 405 nm absorbance using a BioTek ELx800 microplate reader (USA).

### Western blot analysis

SKOV3 cells were seeded onto 6-well plates and pre-cultured for 24 h before treatment. Then, 80 μg/mL PAA or 80 μg/mL PAA + 2 μM MHY1485 was added to the culture medium for 6-h treatment. Cell culture media were discarded and cells were washed two times with cold PBS. Then, 100 μL of RIPA lysis buffer (BIYUNTIAN, China) containing PMSF and M5 protease inhibitor cocktail (M5, BIYUNTIAN) was added to each well to lyse the cells, with gentle shaking for 30 min. The cell lysates were collected in 1.5-mL tubes and were centrifuged at 13,000 *g* at 4°C for 1 min to get rid of the cell debris. The supernatant was collected as total soluble proteins and a bicinchoninic acid assay kit (Solarbio) was employed to detect the concentration of total protein. Protein samples were mixed with 2x SDS loading buffer and boiled at 95°C for 5 min. Then, 10 µg of proteins of each sample was subsequently separated using SDS-PAGE and the separated proteins were transferred to a PVDF membrane at 100 V for 100 min. The membrane was blocked with 5% skimmed milk in PBST at 4°C for 2 h and incubated overnight with the following primary antibodies at 1:1000 dilution in PBST at 4°C: mTOR (ab32028, Abcam), p-mTOR (ab109268, Abcam), p70s6k (#2708, Cell Signaling Technology, USA), p-p70s6k (#9205, Cell Signaling Technology), Bcl-2 (ab32124, Abcam), Bax (ab32503, Abcam), and GAPDH (ab18245, Abcam). After washing 3 times with PBST buffer, the membrane was further incubated with secondary antibodies conjugated with HRP (ab205719, 1:2000, Abcam Inc.) at room temperature for 1 h. Protein band development was performed using BeyoECL Plus reagent (BIYUNTIAN). The bands were imaged using a ChemiDoc MP imaging system (Bio-Rad, USA) and quantified using ImageJ software, with GAPDH serving as a loading control.

### Xenograft tumorigenesis assay

A total of 5×10^6^ SKOC3 cells were resuspended in 200 μL of PBS and inoculated subcutaneously into female nude mice. The length (a) and width (b) of tumors were measured with an electronic Vernier caliper every other day and tumor volume (V) was calculated using the formula: V = a^2^ × b × 0.4, where b is the longest diameter and a is the shortest diameter. When the tumor volume reached about 100 mm^3^, mice were randomly assigned to the saline group (n=6; mice given saline orally) or the PAA group (n=6; mice orally fed with 10 mg/kg PAA). Tumor volume was measured every week. All mice were euthanized after six weeks and the subcutaneous tumor weight was measured.

### Statistical analysis

Statistical analysis was performed by GraphPad Prism 7 (IBM, USA). All data are reported as means±SD. Statistical significances between two groups were measured using two-tailed *t*-test (paired data) or unpaired *t-*test (unpaired data). Multiple-group comparison was performed by two-way analysis of variance (ANOVA) with Bonferroni *post hoc* test. All experiments were repeated at least three times. Differences with a P<0.05 were considered to be statistically significant.

## Results

### PAA displayed anticancer effects in SKOV3 ovarian cancer cells

**Figure 1:**
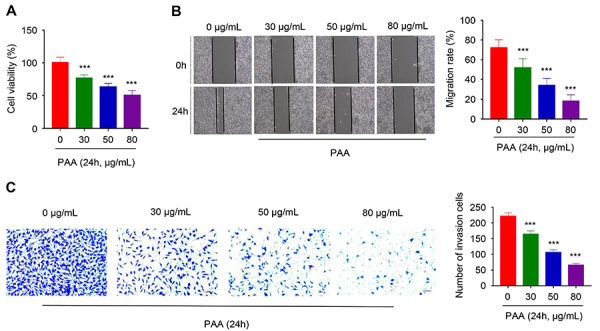
Poricoic acid A (PAA) suppressed the proliferation, migration, and invasion of SKOV3 cells. **A**, CCK-8 assay of cell proliferation on SKOV3 cells treated with 0, 30, 50, and 80 μg/mL PAA for 24 h. **B**, Scratch cell migration assay of cells treated with different concentrations of PAA at 0 and 24 h. **C**, Transwell invasion assay of cells treated with different concentrations of PAA for 24 h (scale bar 50 μm). Data are reported as means±SD of 3 independent experiments. ***P<0.001 compared to Control (0 μg/mL) (two-tailed Student's *t*-test).

We first attempted to explore the anticancer effect of PAA in SKOV3 ovarian cancer cell line with three different concentrations (30, 50, or 80 μg/mL). After 24 h treatment, CCK-8 assay showed that PAA treatment significantly decreased cell viability in a dosage-dependent manner, with a stronger effect at higher concentrations (Figure 1A). We next examined the effect of PAA on migration of SKOV3 cells using the cell scratch assay (would healing assay). We observed that the migration rate of the control group was over 70%, while PAA treatment significantly impaired the migration rate in a dosage-dependent manner, with stronger inhibitory effects at higher concentrations (P<0.001) (Figure 1B). Transwell invasion assay showed similar results with the scratch assay: the number of invading cells decreased with the increasing concentrations of PAA treatment (Figure 1C). These results suggested that PAA treatment suppressed viability and migration in SKOV3 cells.

### PAA induced apoptosis in SKOV3 cells

**Figure 2:**
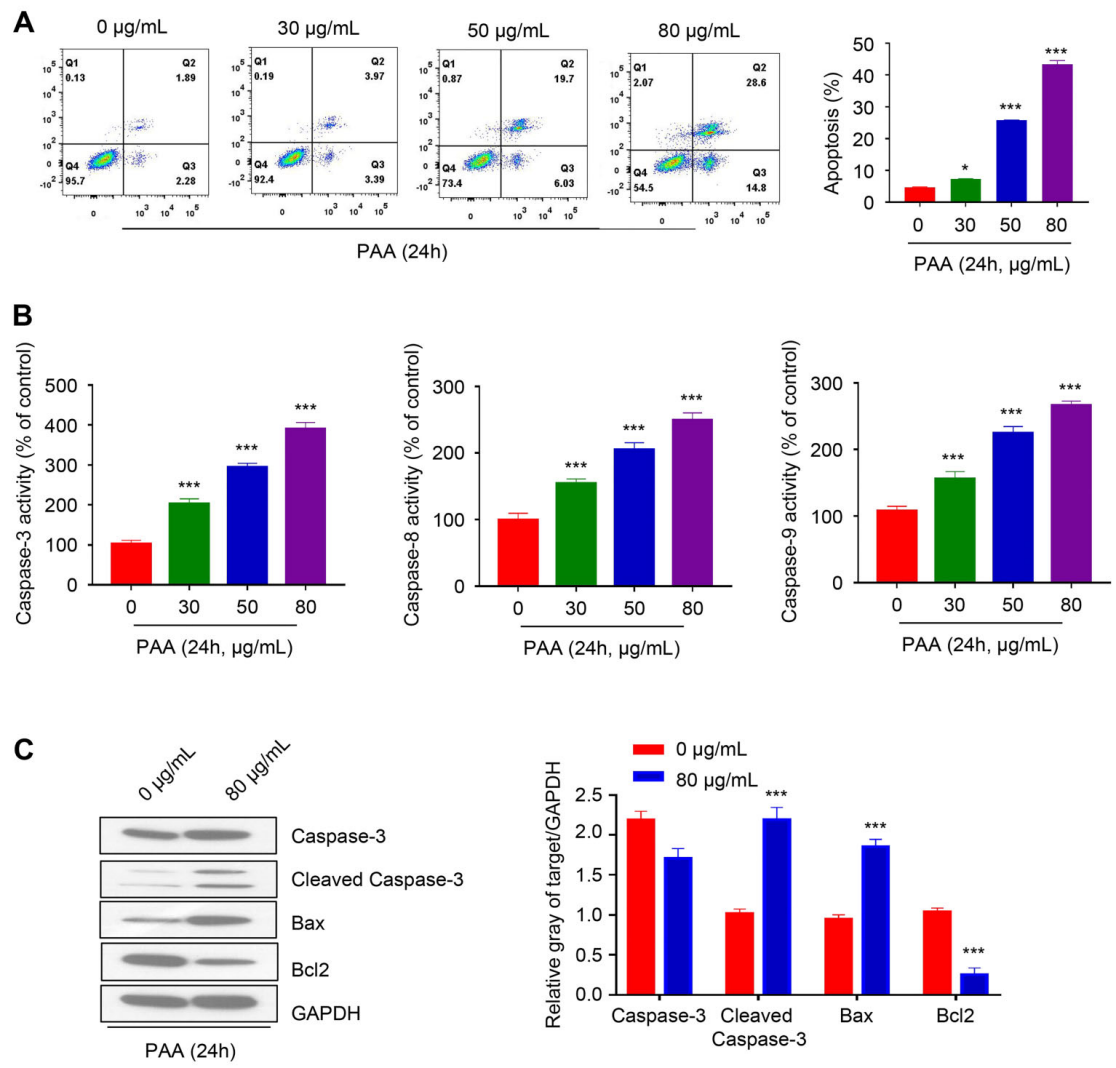
Poricoic acid A (PAA) treatment induced apoptotic cell death. **A**, Flow cytometry analysis of Annexin V-positive cells after treatment with different concentrations of PAA for 24 h. **B**, Colorimetric assay of caspase-3, caspase-8, and caspase-9 activities using total cell lysates from cells treated with different concentrations of PAA for 6 h. **C**, Western blot analysis of caspase-3, cleaved caspase-3, Bax, Bcl2, and GAPDH protein levels in the absence or presence of 80 μg/mL PAA. Data are reported as means±SD of 3 independent experiments. *P<0.05, ***P<0.001 compared to Control (0 μg/mL) (two-tailed Student's *t*-test).

Apoptosis is one of the main cell death processes accounting for anticancer effects of many compounds. The percentage of both early apoptotic cells (Quadrat 3) and late apoptotic cells (Quadrat 4) increased with the increasing concentration of PAA. Overall, at 80 µg/mL, more than 40% of cells were found to be in early or late apoptotic stage (Figure 2A). We further measured the activities of caspase-3/8/9 using caspase activity colorimetric assay after 6-h treatment with PAA. The activity of the untreated group was set as 100% and the levels of the treated groups were normalized to the untreated group. For all 3 caspases, we observed a consistent increase in the enzymatic activities together with the increase of PAA concentration (Figure 2B). Given that PAA at 80 μg/mL showed the strongest apoptosis induction effect, we selected 80 μg/mL as the working concentration in subsequent experiments. At the protein level, PAA treatment led to enhanced cleavage of caspase-3 while the total level of caspase-3 remained comparable between treatment and control groups. We also observed an increase in pre-apoptotic protein Bax while the anti-apoptotic protein Bcl2 level decreased by 70% after PAA treatment (Figure 2C). In summary, our data showed that PAA treatment led to changes in pro- and anti-apoptotic protein expression, accompanied by the activation of apoptotic-executor caspases.

### PAA induced autophagy and inhibited the mTOR/p70S6K signaling pathway

**Figure 3:**
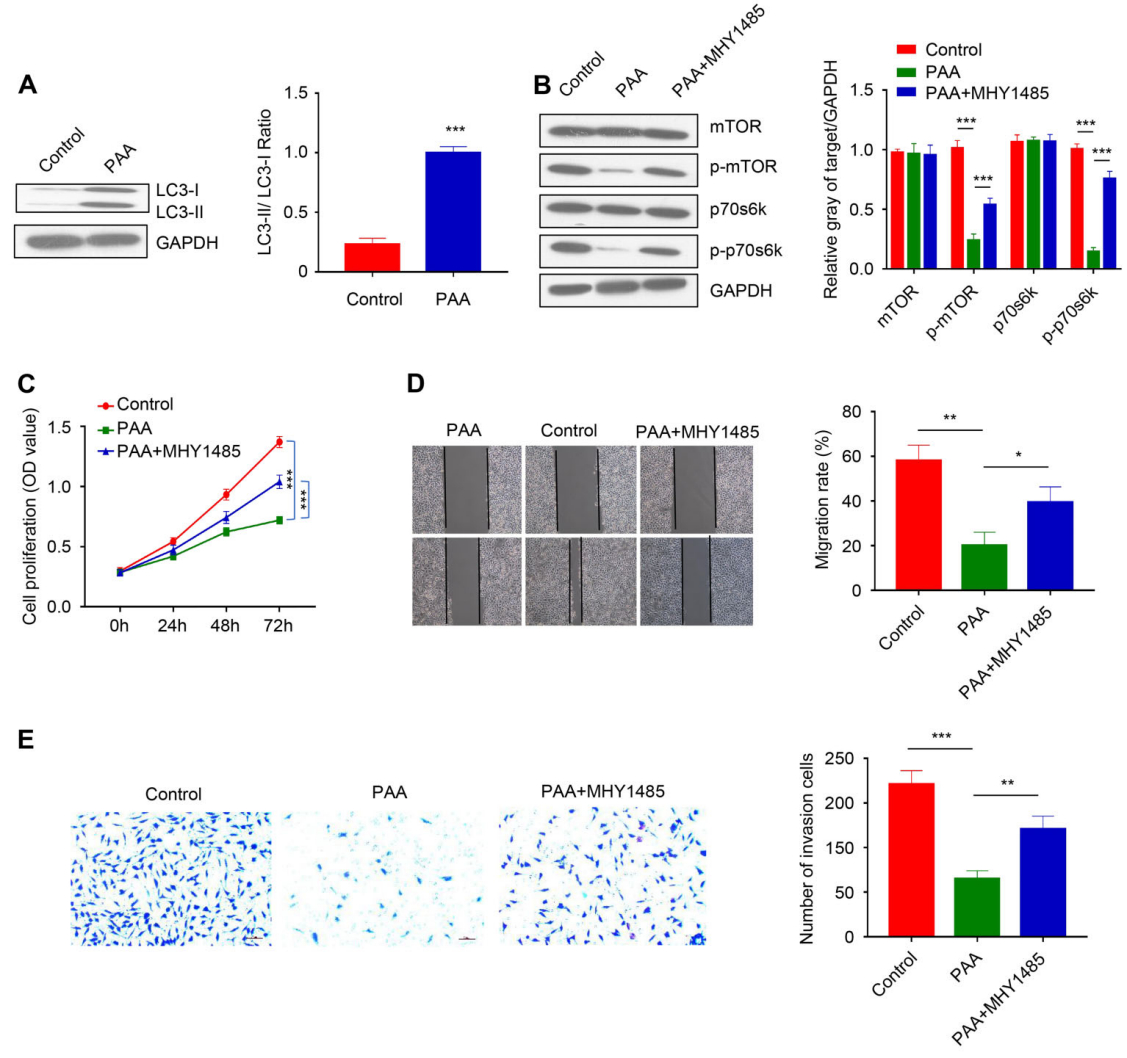
Poricoic acid A (PAA) induced autophagy and suppressed mTOR/p70S6K signaling pathway. **A**, Western blotting analysis of LC3II and LC3I protein level in control and PAA-treated cells. **B**, Western blotting analysis of mTOR, p-mTOR, p70s6k, and p-p70s6k levels in control, PAA-treated cells, and cells with PAA and MHY1485 treatment. **C**, CCK-8 cell proliferation assay in control, PAA-treated cells, and cells treated with PAA and MHY1485 at 0, 24, 48, 72 h. **D**, Scratch assay in control, cells with PAA treatment, and cells with PAA and MHY1485 treatment. **E**, Cell invasion assay in control, cells with PAA treatment, and cells with PAA and MHY1485 treatment (scale bar 50 μm). Data are reported as means±SD of 3 independent experiments. ***P<0.001, **P<0.01, *P<0.05 (two-tailed Student's *t*-test).

PAA treatment not only upregulated LC3-I and LC3-II protein levels, but it also significantly increased the LC3-II/LC3-I ratio (Figure 3A), indicating the imitation of autophagy. It is well known that mTOR signaling pathway is involved in autophagy, apoptosis, and lysosomes formation. Therefore, we next tested the hypothesis that PAA may regulate apoptosis and autophagy by modulating mTOR/p70S6K signaling pathways.

Western blotting results showed that total mTOR and p70s6k levels were not affected by PAA treatment, however, PAA treatment significantly impaired the phosphorylation of mTOR and p70s6k, as detected by antibodies against p-mTOR and p-p70s6k (Figure 3B). The mTOR activator MHY1485, which blocks autophagy by upregulating p-mTOR and downregulating LC3 and p63 expression (14), could partially reverse the inhibitory effect of PAA on mTOR and p70s6k phosphorylation (Figure 3B). The addition of MHY1485 also alleviated the inhibitory effect of PAA on cell proliferation as detected by CCK-8 assay (Figure 3C). Furthermore, cell scratch assay and Transwell migration assay demonstrated that MHY1485 partially attenuated the inhibition of PAA on cell migration and invasion (Figure 3D and E). Collectively, the above results indicated that PAA may exert the anticancer effect in ovarian cancer cells by inhibiting the activity of the mTOR/p70S6K signal pathway. The mTOR agonist MHY1485, which partially restored mTOR/p70S6K phosphorylation, could mitigate the inhibitory effect of PAA treatment.

### PAA treatment suppressed xenograft tumor progression *in vivo*

**Figure 4:**
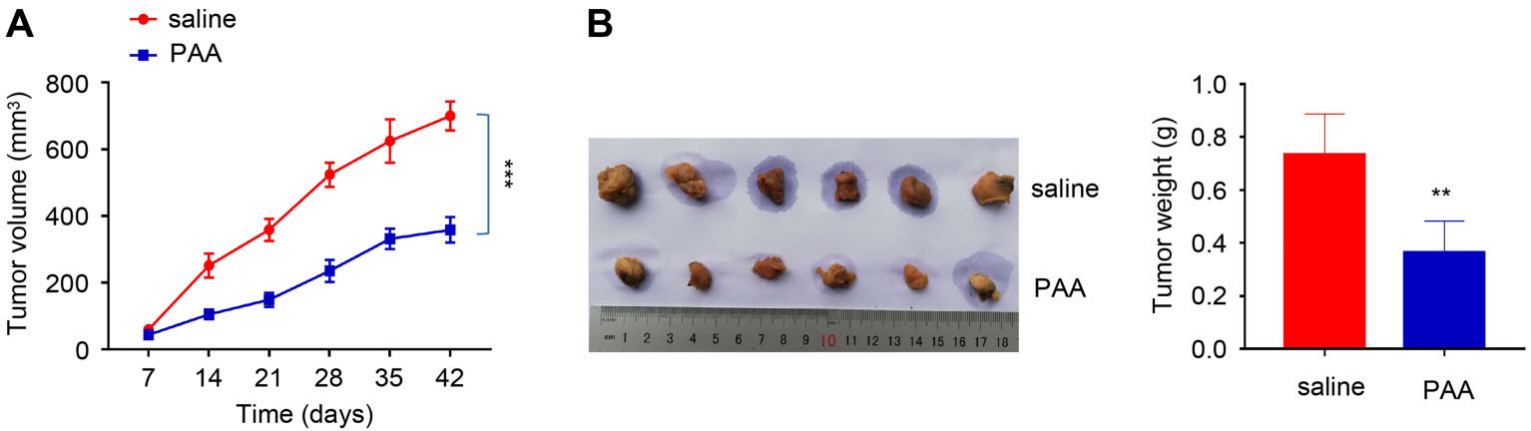
Poricoic acid A (PAA) treatment showed anti-tumorigenesis effects in xenograft model. **A**, SKOV3 cells were injected subcutaneously into female nude mice. The tumor volumes in mice fed with saline and mice with daily PAA intake were measured at 7, 14, 21, 28, 35, 42 days (n=6). ***P<0.001, two-way ANOVA with Bonferroni post hoc test. **B**, Mice were sacrificed on day 42 and the tumor weight was compared between saline and PAA treatment groups. Data are reported as means±SD. **P<0.01, Student's *t*-test.

Tumor size was delayed in the presence of PAA treatment (Figure 4A). After 42 days, mice were sacrificed and we measured the weight of the tumor in each group. PAA treatment reduced the tumor weight by nearly 50% (Figure 4B). Together, our data revealed *in vitro* anticancer effects of PAA in ovarian cancer cells including anti-proliferation, as well as the inhibition of cell migration. PAA treatment also effectively curbed the tumorigenesis and reduced the tumor burden in the *in vivo* xenograft model.

## Discussion

The most common anticancer effects by chemotherapeutics is to induce autophagy or apoptosis in cancer cells. However, there are several issues undermining the treatment outcome of anticancer drugs. First, nearly all types of cancer cells could eventually develop mechanisms to escape cell death or gain drug-resistance, impairing the efficacy of anticancer drugs. Second, many therapeutics elicit anticancer effects by targeting similar biological processes. Resistance to one therapeutic may render the related drugs ineffective. Furthermore, most of the conventional therapeutics also impose toxicity to normal cells especially those in fast cell cycle (15). Traditional Chinese herbal drugs, such as pseudo-ginseng, *Ganoderma lucidum*, ginseng, are well known for their low toxicity and promising anticancer effect (16). However, the mechanisms underlying anticancer effects of traditional Chinese herbal drugs could be complicated since they usually contain multiple bioactive components. Multiple modes of action have been linked to different Chinese medicines, including apoptosis induction, autophagy induction, antiproliferation, and suppressing of DNA duplication (16). Since *Poria cocos* harbors different kinds of bioactive compounds (17-19), it is important to assess the anticancer effects of different components.

In our study, PAA (one major component from *Poria cocos*) greatly suppressed cell proliferation, migration, and invasion in ovarian cancer cells. These effects were linked with the ability of PAA to induce apoptosis and autophagy. Our results are consistent with previous studies, in which *Poria cocos* extracts showed anticancer effects against multiple cancer cells including breast cancer, gastric cancer, and lung carcinoma, both *in vitro* and *in vivo* (17). However, most of previous studies focused on the polysaccharide components. Previous studies have shown that PAA could mildly suppress the growth of different types of cancer cells, including leukemia (HL60), lung (A549), melanoma (CRL1579), breast (SK-BR-3), prostate (DU145), stomach (AZ521), and pancreas (PANC-1). In addition, PAA could induce typical apoptotic cell death in HL60 and A549 cells. In HL60 cells, PAA induces apoptosis through both mitochondrial and death receptor pathways accompanied with increased caspase-3, caspase-8, and caspase-9 expression levels and elevated Bax/Bcl-2 ratio. In contrast, in A549 cells, PAA does not seem to affect caspases-3/8/9 levels, but it enhances the translocation of apoptosis-inducing factor (AIF) and increased Bax/Bcl-2 ratio (18,19). In SKOV3 ovarian cancer cells, our data revealed that PAA induced cell apoptosis and autophagy by suppressing the phosphorylation of mTOR and p70s6k protein, which may downregulate the pro-survival signals regulated by the mTOR/p70s6k axis.

It is well known that autophagy and apoptosis could result from similar inducing signals and they share several common regulatory elements. Akt/mTOR pathway is one of these elements that is commonly dysregulated in cancer cells (20,21). mTOR signaling cascade could be activated by AKT through the inhibition of tuberous sclerosis 1 (TSC1) and tuberous sclerosis 2 (TSC2) expression (22). mTOR signaling pathway is comprised of two complexes, mTOR complex 1 (mTORC1) and mTOR complex 2 (mTORC2). mTORC1 functions to activate ribosomal S6 protein kinase 1 (S6K1) phosphorylation, which in turn activates S6 complex consisting of SKAR, CBP80, PDCD4, eIF4B, and eEF2K (22-24). A number of previous studies demonstrated the involvement of Akt/mTOR pathway in the anticancer effect of PAA (25-27). Our data showed that PAA significantly lowered the phosphorylation level of mTOR and p70s6K, without affecting the total protein level. Importantly, mTOR agonist, which rescues phosphorylation of mTOR and p70s6K, also attenuated the anticancer effect of PAA treatment. Collectively, our data indicated that the apoptosis induction and anticancer effects of PAA in SKOV3 cells could result from its inhibitory actions on the mTOR and p70s6K signaling axis.

In summary, our study demonstrated the anticancer effect of PAA in SKOV3 ovarian cancer cells. Our data also suggested that PAA treatment seemed to negatively affect the phosphorylation of mTOR and p70s6k signaling pathways. Future studies are required to investigate the combinatory effects of PAA with other chemotherapeutics on different types of cancers.

## References

[1] Bray F, Ferlay J, Soerjomataram I, Siegel RL, Torre LA, Jemal A (2018). Global cancer statistics 2018: GLOBOCAN estimates of incidence and mortality worldwide for 36 cancers in 185 countries. CA Cancer J Clin.

[2] American Cancer Society (2008). Cancer facts & figures.

[3] Roett MA, Evans P (2009). Ovarian cancer: an overview. Am Fam Physician.

[4] Webb PM, Jordan SJ (2017). Epidemiology of epithelial ovarian cancer. Best Pract Res Clin Obstet Gynaecol.

[5] Wang YZ, Zhang J, Zhao YL, Li T, Shen T, Li JQ (2013). Mycology, cultivation, traditional uses, phytochemistry and pharmacology of *Wolfiporia cocos* (Schwein.) Ryvarden et Gilb.: a review. J Ethnopharmacol.

[6] Li X, Ma L, Zhang L (2019). Molecular basis for *Poria cocos* mushroom polysaccharide used as an antitumor drug in China. Prog Mol Biol Transl Sci.

[7] Li X, He Y, Zeng P, Liu Y, Zhang M, Hao C (2019). Molecular basis for *Poria cocos* mushroom polysaccharide used as an antitumour drug in China. J Cell Mol Med.

[8] Jeong JW, Lee WS, Go SI, Nagappan A, Baek JY, Lee JD (2015). Pachymic acid induces apoptosis of EJ bladder cancer cells by DR5 Up-regulation, ROS generation, modulation of Bcl-2 and IAP family members. Phytother Res.

[9] Ríos JL (2011). Chemical constituents and pharmacological properties of *Poria cocos*. Planta Med.

[10] Liu X, Yu X, Xu X, Zhang X, Zhang X (2018). The protective effects of *Poria cocos*-derived polysaccharide CMP33 against IBD in mice and its molecular mechanism. Food Funct.

[11] Tian H, Liu Z, Pu Y, Bao Y (2019). Immunomodulatory effects exerted by Poria Cocos polysaccharides via TLR4/TRAF6/NF-κB signaling *in vitro* and *in vivo*. Biomed Pharmacother.

[B12] Dai B, Wu Q, Zeng C, Zhang J, Cao L, Xiao Z (2016). The effect of Liuwei Dihuang decoction on PI3K/Akt signaling pathway in liver of type 2 diabetes mellitus (T2DM) rats with insulin resistance. J Ethnopharmacol.

[13] Lin TY, Lu MK, Chang CC (2020). Structural identification of a fucose-containing 1,3-β-mannoglucan from *Poria cocos* and its anti-lung cancer CL1-5 cells migration via inhibition of TGFβR-mediated signaling. Int J Biol Macromol.

[14] Yeon JC, Park YJ, Park JY, Jeong HO, Kim DH, Ha YM (2012). Inhibitory effect of mTOR activator MHY1485 on autophagy: suppression of lysosomal fusion. PLoS One.

[15] Gewirtz DA (2014). The four faces of autophagy: implications for cancer therapy. Cancer Res.

[16] Singh SS, Vats S, Chia AYQ, Tan TZ, Deng S, Ong MS (2018). Dual role of autophagy in hallmarks of cancer. Oncogene.

[17] Bingshu Li, Jiang Yang, Li Hong, Jianming Tang, Qiannan Li, Qiong Fu (2017). Paeonol induces apoptosis of ovarian cancer cells through the AKT/GSK-3β signaling pathway. Int J Clin Exp Med.

[B18] Zhang L, Tao L, Shi T, Zhang F, Sheng X, Cao Y (2015). Paeonol inhibits B16F10 melanoma metastasis *in vitro* and *in vivo* via disrupting proinflammatory cytokines-mediated NF-kappaB and STAT3 pathways. IUBMB Life.

[19] Chen C, Jia F, Hou Z, Ruan S, Lu Q (2017). Delivery of paeonol by nanoparticles enhances its *in vitro* and *in vivo* antitumor effects. Int J Nanomedicine.

[20] Jung CH, Ro SH, Cao J, Otto NM, Kim DH (2010). mTOR regulation of autophagy. FEBS Lett.

[21] Kumar D, Shankar S, Srivastava RK (2014). Rottlerin induces autophagy and apoptosis in prostate cancer stem cells via PI3K/Akt/mTOR signaling pathway. Cancer Lett.

[22] Kim SH, Son KM, Kim KY, Yu SN, Park SG, Kim YW (2017). Deoxypodophyllotoxin induces cytoprotective autophagy against apoptosis via inhibition of PI3K/AKT/mTOR pathway in osteosarcoma U2OS cells. Pharmacol Rep.

[B23] Sun Y, Huang YH, Huang FY, Mei WL, Liu Q, Wang CC (2018). 3'-Epi-12beta-hydroxyfroside, a new cardenolide, induces cytoprotective autophagy via blocking the Hsp90/Akt/mTOR axis in lung cancer cells. Theranostics.

[24] Zhang DM, Liu JS, Deng LJ, Chen MF, Yiu A, Cao HH (2013). Arenobufagin, a natural bufadienolide from toad venom, induces apoptosis and autophagy in human hepatocellular carcinoma cells through inhibition of PI3K/Akt/mTOR pathway. Carcinogenesis.

[25] Kim SA, Lee HJ, Ahn KS, Lee HJ, Lee EO, Ahn KS (2009). Paeonol exerts anti-angiogenic and anti-metastatic activities through downmodulation of Akt activation and inactivation of matrix metalloproteinases. Biol Pharm Bull.

[B26] Lei Y, Li HX, Jin WS, Peng WR, Zhang CJ, Bu LJ (2013). The radiosensitizing effect of Paeonol on lung adenocarcinoma by augmentation of radiation-induced apoptosis and inhibition of the PI3K/Akt pathway. Int J Radiat Biol.

[27] Tang J, Li B, Liu C, Li Y, Li Q, Wang L (2017). Mechanism of mechanical trauma-induced extracellular matrix remodeling of fibroblasts in association with Nrf2/ARE signaling suppression mediating TGF-beta1/Smad3 signaling inhibition. Oxid Med Cell Longev.

